# Supplementary material for: Assessment of the TRX2p-yEGFP Biosensor to Monitor the Redox Response of an Industrial Xylose-Fermenting Saccharomyces cerevisiae Strain during Propagation and Fermentation
Source: J Fungi (Basel). 2023 May 30;9(6):630. doi: 10.3390/jof9060630 (PMC10300852; doi:10.3390/jof9060630)
Supplement: Supplementary file 1 [file jof-09-00630-s001.zip › jof-2407814-supplementary.pdf]

Supplementary materials

Table S1 Yeast strains used in the present study.

Name	Relevant genotype	Reference
cv-110	MATa URA3 HIS3 LEU2 TRP1 MAL2-8c SUC2	
cv-100+Cas9	cv-110; pCfB2312	This study
TMBRP011	cv-110+Cas9; XI-3::TRX2_5xUASp-yEGFP-ADH1t	This study

Table S2 Plasmids used in the present study.

Name	Relevant genotype	Reference
pCfB2312	pTEF1p-Cas9-CYC1t_kanMX	(Jessop-Fabre et al., 2016)
pCfB3045	gRNA_XI-3; natMX	(Jessop-Fabre et al., 2016)
pCfB2904	XI-3 MarkerFree backbone	(Jessop-Fabre et al., 2016)
pRP005	pCfB2904; TEF1p-yEGFP3-ADH1t	(Perruca-Foncillas et al., 2022)
pRP010	pCfB2904; TRX2_5xUASp-yEGFP-ADH1t	This study

Table S3 List of primers used in the project. Bold letters indicate the introduction of a restriction site. Lower case letters indicate the segment annealing to a gene whereas upper case letters correspond to primer tails.

**Primer name**	**Sequence (5' → 3')**
TRX2p_f_PstI	TACAAAC**CTGCAG**GTATtgtcaacaacgtatctaccaacg
TRX2p_r_XhoI	ATGCGT**CTCGAG**TTAttattgatgtgttatttaaagatatcgtagac
seqTRX2p	aattgggacaacaccagtg
XI-3_ver_r	cggttgtgatattgttcctgc
XI-3_ver_f	ggccgttatttgtgcttgat

Table S4 Volumetric ethanol productivities (g/L/h) obtained for anaerobic fermentations with wheat straw hydrolysate containing levels of inhibitors equivalent to 7.5% WIS, 5% WIS and 2.5% WIS.

	Volumetric ethanol productivity (g/L/h)				
	8 h	24 h	48 h	72 h	96 h
7.5% WISeq GX	1.58±0.11	1.90±0.17	2.35±0.09	2.53±0.09	2.53±0.14
7.5% WISeq H	1.76±0.1	2.27±0.15	2.47±0.14	2.54±0.14	2.55±0.11
7.5% WISeq GE	1.30±0.16	1.89±0.12	2.37±0.08	2.53±0.05	2.59±0.09
5% WISeq GX	1.62±0.12	2.20±0.11	2.44±0.02	2.49±0.0	2.48±0.04
5% WISeq H	1.89±0.11	2.36±0.08	2.24±0.24	2.46±0.05	2.46±0.06
5% WISeq GE	1.68±0.01	2.32±0.03	2.41±0.01	2.46±0.01	2.49±0.03
2.5% WISeq GX	1.81±0.12	2.40±0.07	2.40±0.05	2.45±0.07	2.41±0.11
2.5% WISeq H	1.79±0.26	2.38±0.06	2.84±0.7	2.71±0.49	2.35±0.09
2.5% WISeq GE	1.71±0.35	2.38±0.05	2.79±0.56	2.71±0.43	2.36±0.07

Table S5 Ethanol yields as percentage of the maximum theoretical yield (%max) obtained for anaerobic fermentations with wheat straw hydrolysate containing levels of inhibitors equivalent to 7.5% WIS, 5% WIS and 2.5% WIS.

	Ethanol yield (%max)		
	48 h	72 h	96 h
7.5% WISeq GX	85,4±1.0	92.2±1.3	92±0.6
7.5% WISeq H	89.5±4.4	91.9±4.4	92.3±3.5
7.5% WISeq GE	82.4±2.1	88.1±0.9	90±2.4
5% WISeq GX	87.6±1.2	89.3±2.1	89±0.5
5% WISeq H	80.7±10.5	88.5±0.2	88.5±0.2
5% WISeq GE	86.6±0.7	88.4±1.4	89.5±0.2
2.5% WISeq GX	85.4±3.9	86.9±0.1	85.6±1.6
2.5% WISeq H	102.8±25	98.1±17.3	84.9±2.9
2.5% WISeq GE	98.5±17.5	95.8±13.0	83.8±0.4

Table S6 Percentage of robust coefficient of variance (rCV) of fluorescence intensity during fermentation. Samples with rCV higher than 60% are marked in gray for better visualization.

	Time (h)	1	3	5	8	24	48	72	96
**GX propagation**	10% WISeq	41.7	51.2	63.2	103.15	43.35	45.2	48.75	52.7
	7.5% WISeq	35.75	29.4	30.7	32.4	40.7	45.75	49.7	53.7
	5% WISeq	33.15	28.45	30.25	34.65	47.25	49.65	54.75	52.55
	2.5%WISeq	37.95	38.15	39.3	38.3	50.4	56.7	48.45	42.2
**H propagation**	10% WISeq	58.35	62.45	119.15	55.6	57.35	56.5	56	53
	7.5% WISeq	55.15	43.1	42.3	45.95	56.65	56.85	53.75	52.8
	5% WISeq	52.25	44.1	46.05	50.95	56.25	53.4	49.5	49.65
	2.5%WISeq	53.25	52.2	51.65	52.85	53.75	51.55	46.1	42
**GE propagation**	10% WISeq	45.6	53.2	59.05	74.65	59.15	46.3	47.35	52.15
	7.5% WISeq	45.55	55.05	44.7	48.85	42.75	46.75	59.4	61.5
	5% WISeq	43.7	29.95	40.1	40.75	43.65	48.05	61.05	65.25
	2.5%WISeq	49.15	51.85	49.35	46.1	44.7	66.5	58.15	50.7

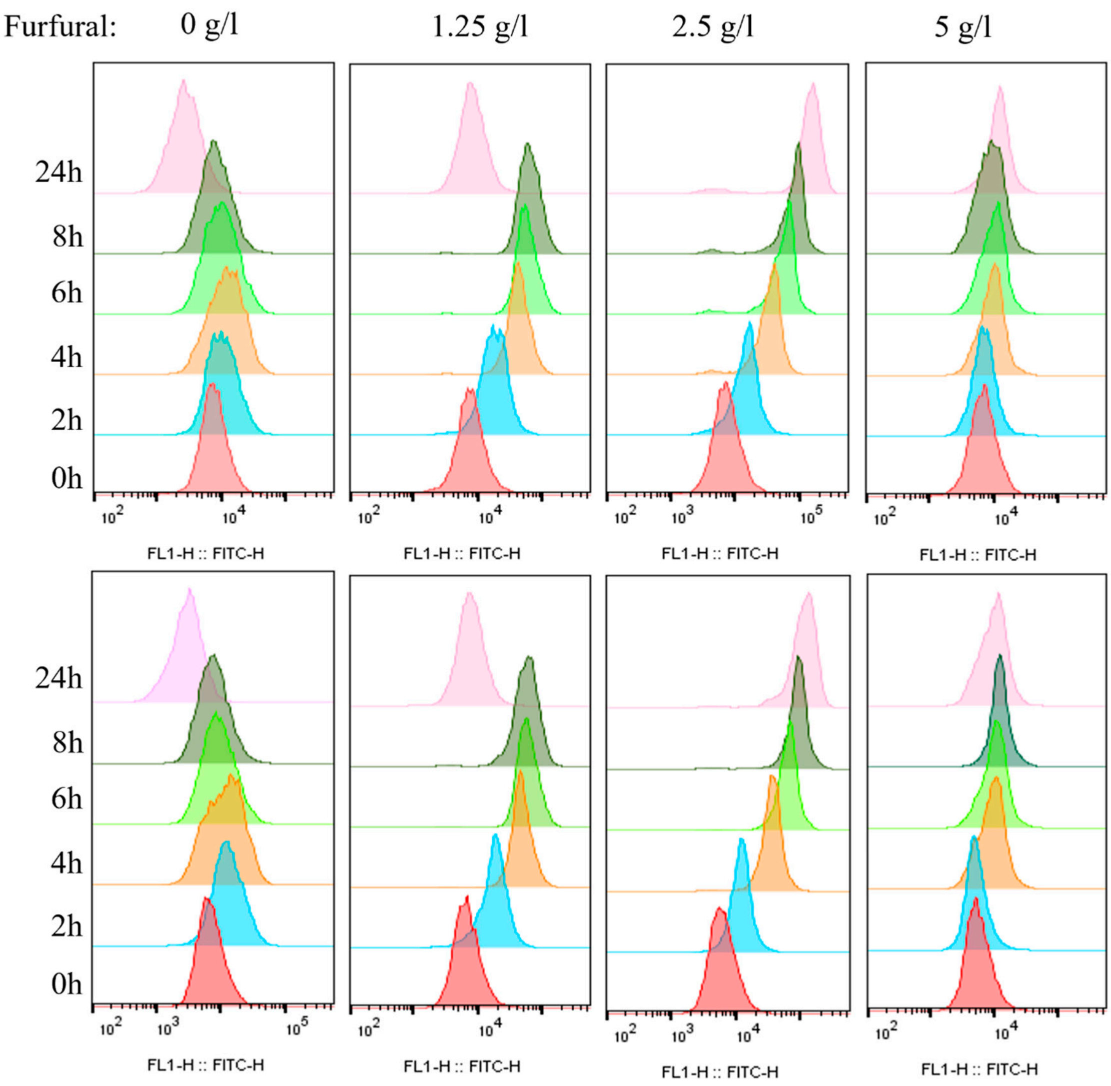

Fig. S1 Histogram showing population distribution of GFP signal from biosensor when exposed to different concentrations of furfural. Each row corresponds to one of the biological replicates.

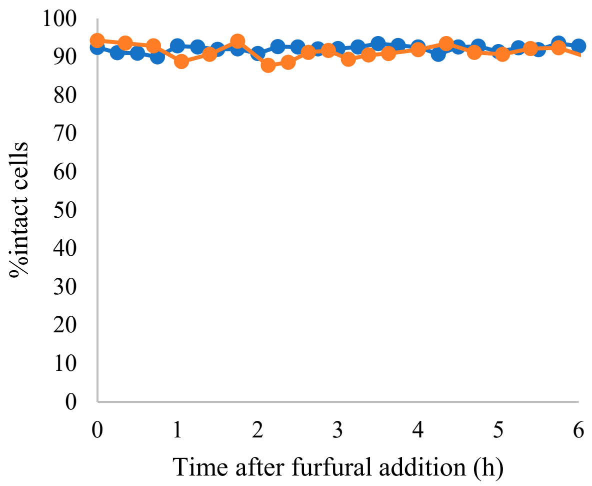

Fig. S2 Percentage of population of cells with intact membranes based on propidium iodine (PI) staining during the first 6 hours after the addition of a pulse of 2 g/L of furfural in a chemostat. Each series corresponds to one of the biological replicates.

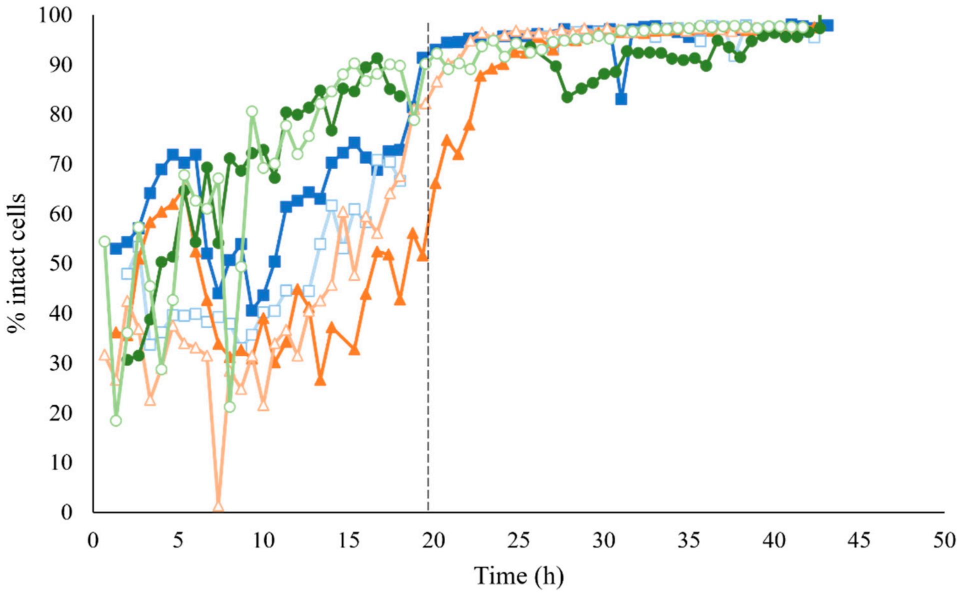

Fig. S3 Percentage of cells in the population with intact membranes based on propidium iodine (PI) staining over time during propagation for three different strategies: H propagation (▲). GX propagation (■) and GE propagation (●). The second biological replicate for each strategy is marked with empty symbols. The dotted line marks the start of the feeding.

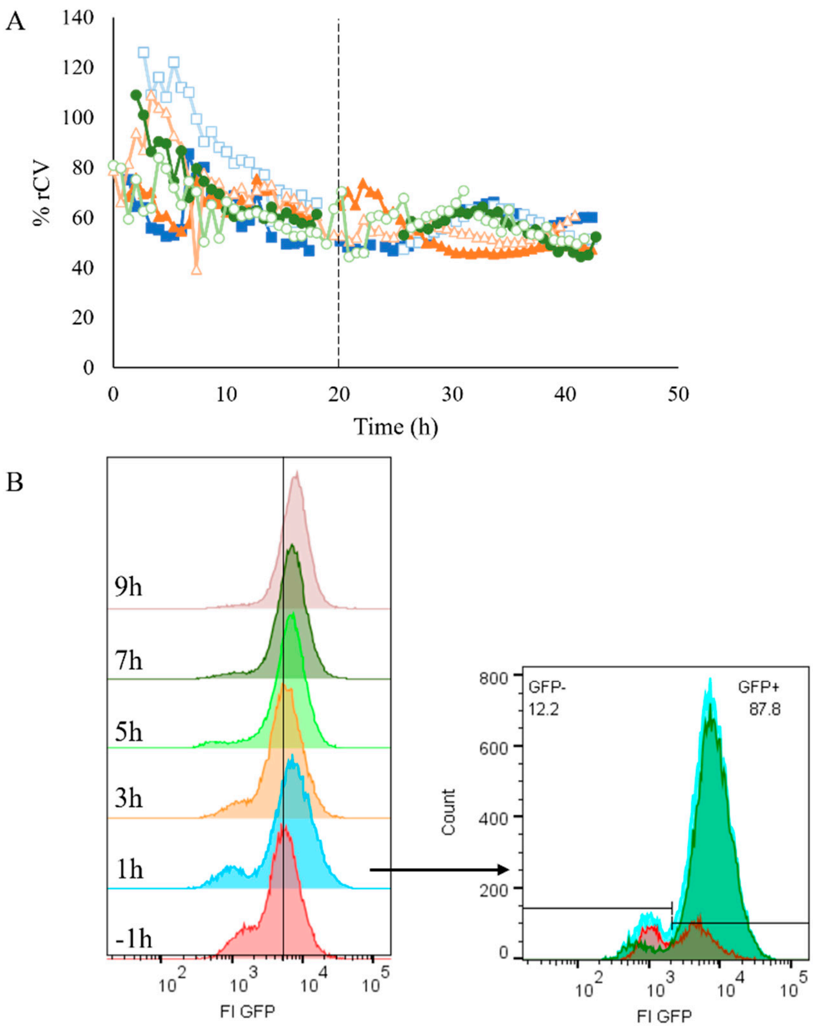

Fig. S4 (A) Robust coefficient of variance (rCV) of fluorescence intensity over time during propagation of three different strategies: H propagation (▲). GX propagation (■) and GE propagation (●). The second biological replicate for each strategy is marked with empty symbols. The dotted line marks the start of the feeding. (B) Histogram showing distribution of fluorescence intensity (FI) in the population for one of the biological replicates during H propagation. On the right. the 1h sample in light blue is further analyzed by showing cells with permeabilized membrane in red and intact cells in green.

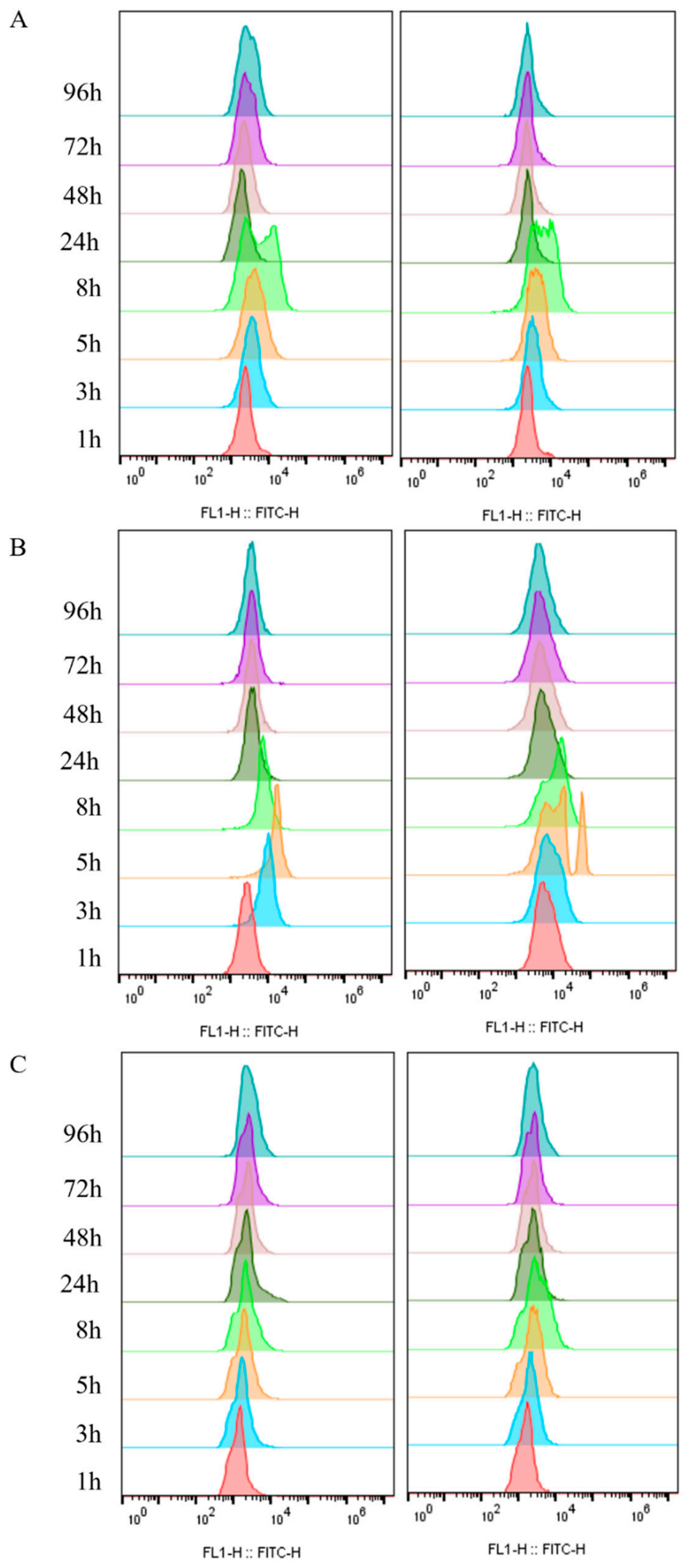

Fig. S5 Histogram representation of distribution of fluorescence intensity (FI) during fermentation at conditions with inhibitors levels equivalent to 10% WIS for (A) GX-propagated cells, (B) H-propagated and (C) GE-propagated cells.
